# CaMKIIα‐TARPγ8 signaling mediates hippocampal synaptic impairment in aging

**DOI:** 10.1111/acel.14349

**Published:** 2024-10-08

**Authors:** Zhao JianHua, MingCan Li, Qilin Hu, Peter Donoghue, Sanwei Jiang, Junmei Li, Songji Li, Xinyi Ren, Ziyuan Zhang, Jingzhi Du, Yi Yu, Paul Chazot, Chengbiao Lu

**Affiliations:** ^1^ Henan International Joint Research Laboratory of Neurorestoratology for Senile Dementia, Henan Key Laboratory of Neurorestoratology, Department of Neurology First Affiliated Hospital of Xinxiang Medical University Weihui, Xinxiang Henan China; ^2^ Henan International Joint Laboratory of Non‐Invasive Neuromodulation, Department of Physiology and Pathophysiology Xinxiang Medical University Xinxiang Henan China; ^3^ Institute of Psychiatry and Neuroscience, Xinxiang Medical University Xinxiang Henan China; ^4^ School of Medical Engineering Xinxiang Medical University Xinxiang China; ^5^ Department of Biosciences Wolfson Research Institute for Health and Wellbeing, Durham University Durham UK

**Keywords:** aging, CaMKIIα, hippocampus, LTP, TARPγ8, VGCC

## Abstract

Aging‐related decline in memory and synaptic function are associated with the dysregulation of calcium homeostasis, attributed to the overexpression of voltage‐gated calcium channels (VGCC). The membrane insertion of AMPAR governed by the AMPAR auxiliary proteins is essential for synaptic transmission and plasticity (LTP). In this study, we demonstrated the hippocampal expression of the transmembrane AMPAR regulatory proteins γ‐8 (TARPγ8) was reduced in aged mice along with the reduced CaMKIIα activity and memory impairment. We further showed that TARPγ8 expression was dependent on CaMKIIα activity. Inhibition of CaMKIIα activity significantly reduced the hippocampal TARPγ8 expression and CA3‐CA1 LTP in young mice to a similar level to that of the aged mice. Furthermore, the knockdown of hippocampal TARPγ8 impaired LTP and memory in young mice, which mimicked the aging‐related changes. We confirmed the enhanced hippocampal VGCC (Cav‐1.3) expression in aged mice and found that inhibition of VGCC activity largely increased both p‐CaMKIIα and TARPγ8 expression in aged mice, whereas inhibition of NMDAR or Calpains had no effect. In addition, we found that the exogenous expression of human TARPγ8 in the hippocampus in aged mice restored LTP and memory function. Collectively, these results indicate that the synaptic and cognitive impairment in aging is associated with the downregulation of CaMKIIα‐TARPγ8 signaling caused by VGCC activation. Our results suggest that TARPγ8 may be a key molecular biomarker for brain aging and that boosting CaMKIIα‐TARPγ8 signaling may be critical for the restoration of synaptic plasticity of aging and aging‐related diseases.

## Graphical abstract

Aging is associated with increased VGCC activity and blunted NMDAR function, which causes dysregulation of calcium homeostasis and impairment of synaptic plasticity (LTP). TARPγ8 expression is dependent on CaMKIIα activity, which is mediated by VGCC activity. The downregulation of CaMKIIα‐TARPγ8 signaling caused by VGCC‐mediated calcium influx results in aging‐related impairment of synaptic plasticity and spatial learning.

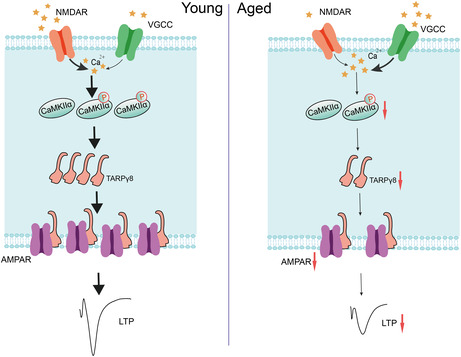

## 1 INTRODUCTION

Normal aging is a major risk factor for age‐related neurodegenerative diseases such as Alzheimer's disease (AD), in which cognitive impairment can be devastating. An important feature of normal aging is learning and memory deficits (Rhodes & Katz, 2017; Uryash et al., 2020), and synaptic impairment (Drulis‐Fajdasz et al., 2015; Tonkikh et al., 2006; Shetty et al., 2017). Long‐term potentiation (LTP), the sustained response of synapses to strong stimuli, has been widely accepted as a cellular model for learning and memory at the synaptic level (Neves et al., 2008) and is associated with altered membrane expression of the ionotropic glutamate receptor channel subtype, α‐amino‐3‐hydroxy‐5‐methyl‐4‐isoxazolpropionic acid receptor (AMPAR) (Lisman et al., 2012). The binding of glutamate to postsynaptic AMPAR mediates rapid excitatory synaptic transmission, essential for neural information processing (Huganir & Nicoll, 2013).

The translocation and function of AMPAR are mediated by various auxiliary subunits (Greger et al., 2017). Among more than 30 AMPAR regulatory proteins (Schwenk et al., 2012; Shanks et al., 2012), transmembrane AMPAR regulatory proteins (TARPs) have attracted wide attention. TARP family members γ‐2, γ‐3, γ‐4, and γ‐8 are classified as type I TARPs, and γ‐5 and γ‐7 belong to type II TARPs (Kato et al., 2008; Soto et al., 2009; Studniarczyk, Coombs, et al., 2013). These TARP family members show differential expression patterns in the brain (Fukaya et al., 2005; Tomita et al., 2003). TARPγ8 is enriched in the hippocampus which controls assemblies, surface trafficking, and gating of AMPAR and plays a key role in synaptic transmission and LTP (Herguedas et al., 2022; Rouach et al., 2005; Xue et al., 2023).

Studies have shown that Ca^2+^/calmodulin‐dependent kinase IIα (CaMKIIα) mediated TARPγ8 phosphorylation, is critical for LTP induction (Baltaci et al., 2019; Lisman 2017; Lisman et al., 2012; Park, Chavez, et al., 2016). CaMKIIα also regulates the phosphorylation of other TARP family members such as TARPγ2 (Rouach et al., 2005) and AMPARs (Lee et al., 2010).

Aging‐related impairment in cognitive function and LTP is associated with calcium dyshomeostasis. Increased expression of voltage‐gated calcium channel (VGCC) in the brain of aged rodents contributes to the abnormal increase of intracellular calcium concentration ([Ca^2+^]_i_) and the activation of small conductance calcium‐dependent potassium channel, resulting in blunted NMDAR function and the increased threshold of LTP induction (Cheng et al., 2013; Guo et al., 2017; Ris & Godaux, 2007).

Aging‐related increase in [Ca^2+^]_i_ may also cause activation of a series of calcium‐dependent proteins such as calcium protease (calpain), a calcium‐dependent cysteine protease. The calpain isoforms in the brain include m‐calpain (calpain‐2) and μ‐calpain (calpain‐1), which are activated by millimolar and micromolar concentrations of calcium, respectively (Tremper‐Wells & Vallano, 2005a). Calpain inhibitors restore synaptic function and memory in a mouse model of AD (Trinchese et al., 2008), suggesting that calpain involvement in AD‐related impairment of synaptic function. In cultured neurons, we found that calpain activity‐dependently downregulated TARPγ8 expression (Wang et al., 2016b). Therefore, we proposed that aging‐related LTP impairment may be related to the reduced CaMKIIα—TARPγ8 phosphorylation and /or the increased calpain‐mediated degradation of TARPγ8.

## 2 MATERIALS AND METHODS

### 2.1 Animals

All animal use procedures were approved by the Ethics Committees at Xinxiang Medical University for the Care and Use of Laboratory Animals, and all efforts were made to minimize animal suffering and reduce the number of animals used. Electrophysiological studies were performed on hippocampal slices prepared from C57BL/6J mice (male). The young group was selected to be 3–4 months old, while the aging group was selected to be 24–28 months old.

### 2.2 Behavioral study

#### 2.2.1 Y maze

Mouse cognitive function were tested by Y maze, consisting three identical arms (start, novel and third arm, arm size 30 cm × 8 cm × 15 cm) with an angle of 120 degrees. The novel arm closed in the training period and opened in the test period. The start arm is the one where the mouse enters the maze. The start and other arms kept open throughout the experiment, allowing the animal to move in and out freely. During the training period, the mouse was placed in the starting arm and allowed to explore two arms for 5 min. After training, the mouse was returned to its home cage for a 30‐min inter‐trial interval. In the test period, the block in the novel arm was removed, the mouse was again placed into the start arm and then allowed to access all three arms of the maze. At the end of each training or test, the arms were cleaned to prevent odor cues. Time in the novel arm (%) was defined as the time spent in the novel arm divided by the time spent in all arms during the first minute of the retrieval trial.

#### 2.2.2 The elevated plus maze (EPM)

The EPM was used to examine anxiety‐like behaviors. The behavioral apparatus consisted of two open arms (width 5 cm × length 30 cm) and two closed arms (width 5 cm × length 30 cm) elevated 50 cm above the floor for the mice. Mice were placed individually in the center of the maze facing an open arm and allowed to explore for 5 min. The following behavioral parameters were automatically determined: duration and entries into the open arms, closed arms, and center zone and total distance traveled. The percentage of open arm entries (open arm entries/total arm entries) was calculated. The maze was cleaned with 75% ethanol after each test to prevent the influence of previously tested animals.

#### 2.2.3 The open field test (OFT)

The OFT was used to analyze locomotion, anxiety, and stereotypical behaviors. The test was conducted for 10 min in a dimly lit room. The test area normally consisted of a large arena measuring 42 cm × 42 cm × 42 cm. The arena was made of high‐density polyethylene panels that were fastened together and placed on a plastic bottom plate. The mouse behavior was recorded with a camera mounted above the arena. The following behaviors were analyzed: total distance traveled, distance traveled in each zone (outside, middle, and center), number of entries into each zone, and latency to enter the middle and center zone. The maze was cleaned with 75% ethanol after each test to prevent the influence of previously tested animals.

#### 2.2.4 Novel object recognition (NOR) test

NOR test was used to assess memory and exploration ability of mice by utilizing the nature of preferential exploration of the novel object (Leger, Quiedeville, et al., 2013). In the first part of the test (the acquisition phase), two identical objects were placed in two different quarters of the arena and animals were allowed to freely explore the arena and the objects for 5 min. In the subsequent testing phase, one of the similar objects was replaced by a novel object and the animal was again allowed to explore the arena and objects for 5 min. The time between acquisition and testing phase was set to 1 h. The behavior of the mice was recorded with a camera.

Preference index (PI) and the discrimination index (DI) reflects the duration of exploration for the novel object compared to the old object as a proportion of the animal's total exploration time, and is used to quantify the performance of the NOR task, which is assumed to represent recognition memory sensitivity.

The time that mice explored the new object (T_new_) and the old object (T_old_) was measured. PI was calculated as T_new_/(T_new_ + T_old_), and DI was calculated as (T_new_‐T_old_)/(T_new_ + T_old_). To avoid object preference biases, objects A and B were counterbalanced so that one half of the animals in each experimental group were first exposed to object A and then to object B, whereas the other half first saw object B and then object A. The maze and the objects were cleaned with ethanol after each test in order to eliminate olfactory cues.

#### 2.2.5 Morris water maze (MWM) test

MWM test was used to assess the spatial learning and memory of rodents. Briefly, the laboratory mice were first trained for five days of place navigation experiment, and on the seventh day for space exploration experiments. In the first 5 days, an escape platform was placed in the designated quadrant, and the time the mice spent searching for the platform was used as an evaluation index of their learning ability. On the seventh day, after the platform was removed, the time the mice swam in the target quadrant and the number of times they crossed the platform were recorded, which reflected the mice's memory ability. The VisuTrack MWM image analysis system was used for data acquisition and analysis.

### 2.3 Western blotting

The protein from hippocampal tissue was extracted for the standard Western blot analysis. The protein was denatured at 100°C for 5 min and was separated by 10% sodium dodecyl sulfate‐polyacrylamide gel electrophoresis (SDS‐PAGE). Protein was transferred onto PVDF membranes (Millipore, Boston, MA, USA) using a Mini PROTEAN Tetra Cell (Bio‐Rad, Hercules, CA, USA) following the manufacturer's instructions. Transferred membranes were blocked in 5% skim milk dissolved in Tris‐buffered saline pH 7.5/0.1% Tween‐20 (TBST) for 1.5 h at room temperature and then incubated at 4°C overnight with primary antibodies: calpain1 (1:1000, 82 kDa, Abcam, Cat# ab108400, knockout validated), calpain2 (1:2500, 82 kDa, Abcam, Cat# ab126600, knockout validated), TARPγ8 antibody (1:10000, 55 kDa, Frontier Institute), CaMKIIα (1:5000, 54 kDa, Abcam, EPR1828) (ab92332) (Cat#ab92332), p‐CaMKIIα (1:5000, 54 kDa, Abcam, Cat#ab171095), VGCC (1:1000,245 kDa,Abmart, PU127600). The membranes were washed three times with TBST and then incubated with appropriate conjugated hydrogen peroxidase HRP secondary antibody (1:2000, Abcam) for 1 h at room temperature. Proteins were visualized using the enhanced chemiluminescence (ECL) reagent (4A Biotech Co. Ltd., Shanghai, China) and quantified using Image J. The internal control of GAPDH (1:5000, 36 kDa, Proteintech, Cat# 10494‐1‐AP) or β‐actin (1:5000, 42 kDa, Proteintech, Cat# CL594‐60008) was reprobed for each membrane after washing out the target antibody.

### 2.4 IHC‐P (Immunohistochemical analysis of paraffin sections)

Brain tissues from young adult (3–5 months old) and aged (18 months old) mice were first perfused with 0.9% sodium chloride (NaCl) and subsequently fixed with 4% paraformaldehyde. Following fixation, the tissues were washed in 0.1 M phosphate‐buffered saline (PBS, pH 7.4) and dehydrated through a series of ethanol gradients. The dehydrated tissues were then embedded in paraffin, sectioned into 4 μm thick slices, and mounted onto glass slides. The sections were initially heated at 60°C for 30 min, then subjected to deparaffinization using a series of limonene and ethanol solutions. For antigen retrieval, the deparaffinized sections were incubated in 10 mM sodium citrate buffer (containing 0.05% Tween 20, pH 6.0) at 80°C for 1 h, followed by washing with PBS. After these procedures, the sections were incubated with a blocking solution containing 10% goat serum in PBS for 1 h at room temperature. The sections were then incubated overnight at 4°C with the primary antibodies: rabbit L‐type VGCC antibody (1:200, Cav1.3, PU127600, Abmart, China) and rabbit anti‐TARPγ8 (1:200, DF2266, Affinity, USA). The next day, the sections were washed with PBS and incubated with a secondary antibody (HRP‐labeled Cy3 Goat anti‐Rabbit IgG) at room temperature for 30 min in a humidity chamber, followed by another PBS wash. Immunostaining was visualized by incubating the sections in 3,3′‐diaminobenzidine (DAB) (Proteintech, Wuhan, PK10006) as a chromogen. Finally, the sections were counterstained with hematoxylin and mounted. The positive immunostaining for L‐type VGCC and TARPγ8 was evaluated using a microscope (Nikon, ECLIPSE 55i), with nuclei stained blue by hematoxylin and positive DAB expression appearing brownish yellow.

### 2.5 Stereotaxic viral injection

The virus was successfully constructed by Heyuan Biotechnology (Shanghai) Co., Ltd. Target gene: CACNG8 /TARP Gamma 8 NM_133190 [CdS:1272 bp] [Protein: 423AA], target plasmid: H10669 pAAV‐CMV‐EGFP‐2A‐Cacng8‐3FLAG, control plasmid: AOV024 pAAV‐CMV‐EGFP‐2A‐MCS‐3FLAG. The mice were anesthetized by Sagatal (sodium pentobarbitone, 100 mg kg − 1, Rhône Mérieux Ltd., Harlow, UK) and fixed on a stereotaxic device. The skin of the skull was disinfected and cut. After adjusting the top of the skull on a plane, determining the position of CA1 (AP: −1.82 mm relative to bregma, ML: ±1.50 mm from the midline, and DV: −3.0 mm below the dura). Then plasmid (200 nL) was injected on both sides of hippocampi. Finally, the skin was sutured, and anti‐inflammatory drug (neomycin ointment) was applied. Tolfenamic Acid (i.p.) was used as an analgesia in post‐operative pain and fever.

We constructed adeno‐associated virus (AAV) vector containing short hairpin RNA (shRNA) targeted for TARPγ8 to achieve knockdown of Cacng8, we injected pscAAV‐U6‐shRNA(Cacng8)‐cmv‐EGFP‐Twpa (AAV‐Cacng8‐shRNA or TARPγ8‐KD) or pscAAV‐U6‐shRNA(NC2)‐cmv‐EGFP‐Twpa (AAV‐NC‐shRNA, NC: Negative control) viruses into the hippocampal CA1 of 6‐month‐old mice. The expression pattern of AAV‐Cacng8‐shRNA was confirmed by fluorescence imaging and western blot.

### 2.6 Preparation of hippocampal slices

For electrophysiology, the animals were anesthetized by intraperitoneal injection of Sagatal. When all pedal reflexes were abolished, the animals were perfused intracardially with chilled (4°C), oxygenated artificial cerebrospinal fluid (ACSF) in which the sodium chloride had been replaced by iso‐osmotic sucrose. This ACSF (305 mosmol‐1) contained (in mM): 225 sucrose, 3 KCl, 1.25 NaH_2_PO_4_, 24 NaHCO_3_, 6 MgSO_4_, 0.5 CaCl_2_, and 10 glucose. For extracellular field recording, the hippocampal horizontal slices (350 μm) of mouse brains were cut at 4–5°C in the sucrose‐ACSF, using a Leica VT1000S vibratome (Leica Microsystems UK, Milton Keynes, UK). Hippocampal slices were then transferred to an incubation chamber, where they remained submerged in oxygenated ACSF, which consisted of (in mM), 126 NaCl, 3 KCl, 1.25 NaH_2_PO_4_, 2 MgSO_4_, 24 NaHCO_3_, 2 CaCl_2_, and 10 glucose, pH 7.35–7.45 at room temperature until used for recording.

### 2.7 Electrophysiological recordings

After at least 1 h of equilibration, slices were transferred to an interface‐type recording chamber where they were perfused with ACSF of 32°C, at a rate of 3–4 mL/min, with their surface exposed to warm, humidified carbogen (95% O2‐5% CO2). Field potentials were recorded from SR of CA1, using a glass pipette filled with ACSF (resistance was 3–5 MΩ). Recordings were band‐pass filtered online between 0.5 Hz and 2 kHz using an Axoprobe amplifier (Digitimer Ltd., Welwyn Garden City, UK) and a Neurolog system NL106 AC/DC amplifier (Digitimer Ltd., Welwyn Garden City, UK). The data were digitized at a sample rate of 10 kHz using a CED 1401 Plus ADC board (Digitimer Ltd). Electrical interference from the mains supply was online eliminated from extracellular recordings using HumBug noise eliminators (Digitimer Ltd). PSPs were evoked by orthodromic stimulation of the SC‐commissural fibers in CA1 SR, using twisted nickel/chromium wires (50 μm diameter). Pulses of 0.1 ms duration were delivered every 20 s. Pulses were given at varying stimulus intensity to establish a stimulus intensity–response relationship. The downward PSP slope was calculated between 10% and 50% of the maximum amplitude of the PSP. The standard stimulus intensity was set at the intensity that evoked a PSP amplitude 50% of the maximum PSP amplitude. After a stable baseline (15–30 min) was established, long‐term potentiation (LTP) was induced by a single high‐frequency stimulation (HFS, 100 Hz, 1 s), after which changes in the PSP slope were recorded for 60 min. To test the effect of drugs on LTP, drugs were applied to the ACSF 20–40 min before the HFS and were then present throughout the experiment. KN93, calpeptin, DAP5, and nifedipine were purchased from Tocris. All other agents were purchased from Sigma.

### 2.8 Drugs

KN93, calpeptin, DAP‐5, NMDA, forskolin, and nifedipine were purchased from Tocris. All other agents were purchased from Sigma. Stock solutions at a thousand times the final concentration were made up in water or DMSO, and stored in individual aliquots at −20°C. Final solutions were prepared freshly on the day of the experiment. Drugs were applied to the recording ACSF after baseline fEPSP reached a steady state for 15 min and continuously presented in the solution until the recording finished.

### 2.9 Data analysis and statistics

Smart V3.0 software was used to record and analyze behavioral data; Western blotting data was analyzed using ImageJ software; The Spike 2 software was used to record the field potential.

All statistical analyses were performed using IBM SPSS Statistics 22 software (IBM, Armonk, NY, USA). The Shapiro–Wilk test was used in testing the normality of the data. Parametric data were expressed as mean ± standard error of the mean. The paired and unpaired Student's *t*‐tests were used to compare two groups of parametric data.

The unpaired *t*‐test is for the groups from two different populations or independent samples. The paired *t*‐test is for the groups from a single population or for measuring before and after an experimental treatment. One‐way analysis of variance (ANOVA) and repeated measures (RM) ANOVA were used to compare three or more group means. Non‐parametric data were expressed as median ± interquartile range. The Wilcoxon rank‐sum and signed‐rank tests were used to compare groups of non‐parametric data. One‐way and RM ANOVA on Ranks were used for three or more group comparisons of nonparametric data. A *p*‐value <0.05 was considered statistically significant.

## 3 RESULTS

### 3.1 Age‐dependent decline in learning and memory and TARPγ8 expression

**FIGURE 1:**
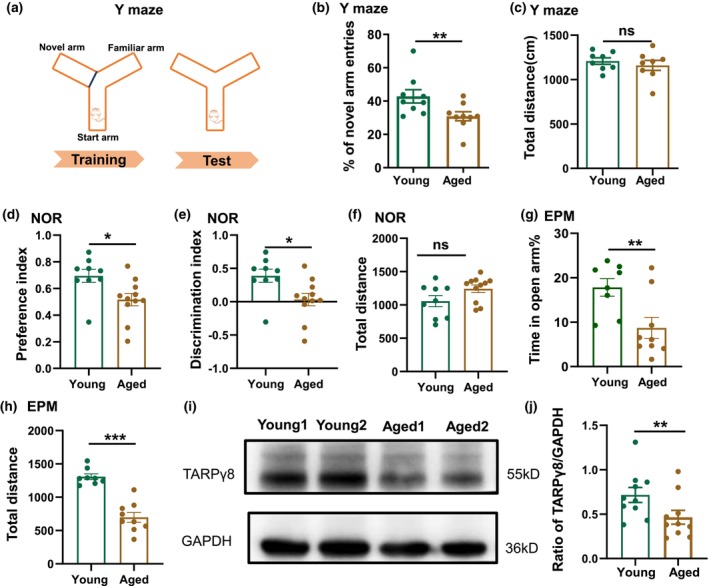
Aging‐related decline in TARPγ8 expression and learning and memory. (a) A diagram of the Y maze experiment, where three arms represent the start arm, the novel arm, and the other arm. (b) The percentage of mice (young and aged) entries into the novel arm in Y maze. (c) The total distance of mice traveled in Y maze. (d) Preference index for the new object in the NOR test, calculated as T_new_/(T_new_ + T_old_). (e) Discrimination index for the new object in the NOR test session, is calculated as (T_new_‐T_old_)/(T_new_ + T_old_). (f) Total distance of mice traveled in NOR. (g) The percentage of time that mice spent in open arm in EPM. (h) Total distance of mice traveled in EPM. (i) The expression of TARPγ8 in the hippocampi of young and aged mice determined by Western Blot analysis. Representative image of TARPγ8 bands (55 kDa) and GAPDH bands (36 kDa) of young and aged mice. (j) The bar graph for the ratio of TARPγ8/GAPDH of young and aging mice. Y: Young mice for 3–4 months old, A: Aged mice for 22–27 months old (young vs. Aged, **p < 0.01, *t*‐test, *n* = 6 mice for each group). Data were expressed as mean ± SEM, *p < 0.05; **p < 0.01; ***p < 0.001.

Y maze was used to study the spatial working memory of mice in this study. Our results show that the percentage of the novel arm entries was significantly higher in young mice than that of aged mice (young: 46 ± 3% vs. aged: 34 ± 4%, *p = 0.037, *t*‐test, *n* = 9 for each group, Figure 1a,b). There was no age‐related difference in the total distance for the mice traveled in the maze (Figure 1c).

NOR test was used to assess mouse memory and exploration ability. Compared with young mice, both PI and DI were decreased in aged mice (PI: young: 0.69 ± 0.05 vs. aged: 0.52 ± 0.05, *p = 0.018, *t*‐test, *n* = 9 and 11 for young and aged mice, respectively, Figure 1d; DI: young: 0.39 ± 0.09 vs. aged: 0.03 ± 0.09, *p = 0.018, *t*‐test, *n* = 9 and 11 for young and aged mice, respectively, Figure 1e). There was no age‐related difference in total distance of the mice traveled (Figure 1f). These results indicate the reduced memory and exploration ability in aged mice.

EPM showed that compared to young mice, aged mice had reduced percentage of time spent in open arm (young: 17.8 ± 2.0% vs. aged: 8.7 ± 2.1%, **p = 0.007, *t*‐test, *n* = 8 and 9 mice for young and aged mice, Figure 1g) and reduced total distance of mice traveled (young: 1307.8 ± 43.7 vs. aged: 698.6 ± 73.6, ***p < 0.001, *t*‐test, *n* = 8 and 9 for young and aged mice, Figure 1h). These results indicated the aged mice had decreased capability in recognition memory and increased anxiety.

Western blotting analysis showed that the expression level of TARPγ8 in the hippocampus of aged mice was significantly lower than that of young mice (young: 0.65 ± 0.06 vs. Aged: 0.41 ± 0.05, **p = 0.01, *t*‐test, *n* = 9 mice for each group; Figure 1i,j). Experiments with immunohistochemistry and western blot showed that TARPγ8 had superior expression in the hippocampus in young mice using tissue samples from different brain regions of mice, but a reduced expression in aged mice (Figure S1). These results showed that the decreased learning and memory functions in aged mice may be associated with decreased expression of TARPγ8.

### 3.2 Age‐related change of expression level of p‐CaMKIIα

**FIGURE 2:**
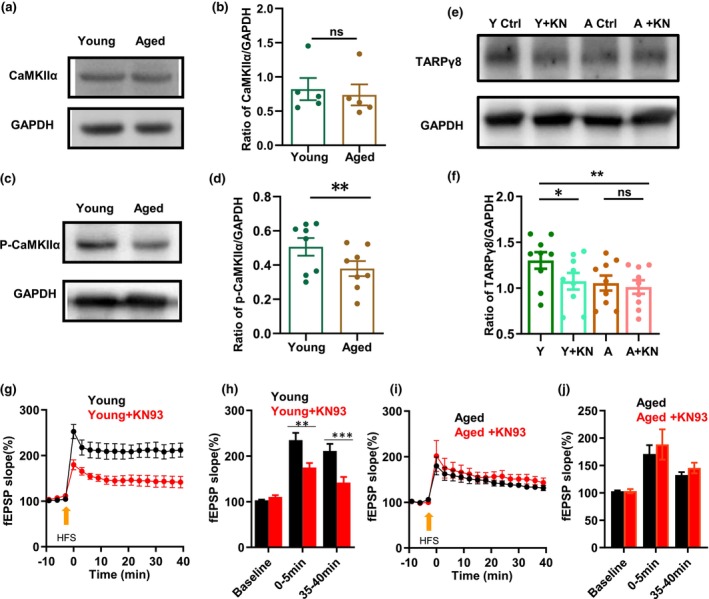
Aging‐related decrease in the expression of p‐CaMKIIα and effect of inhibition of CaMKIIα activity on TARPγ8 expression. (a, b) Western Blot assay showed the expression of CaMKIIα in the hippocampi of young and aged mice. Upper panel: Representative image of CaMKIIα band (50 kDa) and GAPDH bands (36 kDa) of young and aged mice. Low panel: The bar graph for the ratio of CaMKIIα/GAPDH of young and aged mice (Young vs. Aged, *p* = 0.714, *t*‐test, *n* = 5 mice for each group). (c, d) Western Blot assay showed the expression level of p‐CaMKIIα in the hippocampi from young and aged mice. Upper panel: Representative image of p‐CaMKIIα band (50 kDa) and GAPDH bands (36 kDa) of young and aged mice. Low panel: The bar graph for the ratio of p‐CaMKIIα/GAPDH of young and aged mice (young vs. aged, **p = 0.006, *t*‐test, *n* = 8 mice for each group). Y: Young mice for 3–4 months old, A: Aged mice for 22–27 months old. (e) Representative image of TARPγ8 bands (55 kDa) and GAPDH bands (36 kDa) of young and aged mice in the presence or absence of KN93 (20 μM). (f) The bargraph shows the ratio of TARPγ8/GAPDH from young and aged mice (young ctrl vs. Young +KN93, *p = 0.013; Aged ctrl vs. Aged +KN93, *p* = 0.36, One‐way RM ANOVA followed by post hoc Holm‐Sidak method, *n* = 7 mice for each group). Y for 3–4 months old mice, A for 22–27 months old mice. Ctrl: Control (ACSF); KN: KN93. (g, h) The time‐effect curves of the field EPSP (fEPSP) slope before and after HFS in young mice. Histogram gives the HFS‐induced LTP slope for early LTP peak state (average over 0–5 min post HFS) and steady state (average over 35–40 min post HFS), for slices from young mice (*n* = 8 slices from 4 mice) or young + KN93 (*n* = 9 slices from 4 mice). (i, j) The time‐effect curves of the fEPSP slope before and after HFS in aged mice. Histogram gives the HFS‐induced LTP slope for early LTP peak state (average over 0–5 min post HFS) and steady state (average over 35–40 min post HFS), for slices from aged mice (*n* = 10 slices from 4 mice) or aged + KN93 (*n* = 5 slices from 4 mice). Student's *t*‐test was used for comparisons between slices perfused with Ctrl (ACSF) or KN93. Data were given as mean ± SEM, *p < 0.05; **p < 0.01; ***p < 0.001.

It was reported that CaMKII‐mediated phosphorylation of TARPγ8 (Park, Chavez, et al., 2016), we thus examined the expression of both the total level and phosphorylated form of CaMKIIα (p‐CaMKIIα). Compared with the young mice, our results demonstrated that p‐CaMKIIα level was significantly decreased in the hippocampus of aged mice (young: 0.51 ± 0.05 vs. Aged: 0.38 ± 0.04, **p = 0.006, *t*‐test, *n* = 8 for each group, Figure 2c,d), but the total CaMKIIα level was not altered (young: 0.82 ± 0.16 vs. Aged: 0.74 ± 0.15, *p* = 0.714, *t*‐test, *n* = 5 for each group, Figure 2a,b). These results indicate that aging is associated with the reduced CaMKIIα activity.

### 3.3 CaMKIIα activity is required for TARPγ8 expression and LTP

Given the decreased expression of both p‐CaMKIIα and TARPγ8 in aging, there may be an association between TARPγ8 expression and CaMKIIα activity. To investigate the relationship between the expression of TARPγ8 and the activity of CaMKIIα, we analyzed the expression of TARPγ8 in the presence of CaMKIIα inhibitor KN93. We found that KN93 significantly reduced the expression level of TARPγ8 in young but not aged mice (young control: 1.34 ± 0.10 vs. KN93: 1.10 ± 0.1, *p = 0.013; aged ctrl: 1.08 ± 0.08 vs. KN93: 1.10 ± 0.08, *p* = 0.36, One‐way RM ANOVA followed by post hoc Holm‐Sidak method, *n* = 9 mice for each group) (Figure 2e,f). These results showed that the expression of TARPγ8 was dependent on CaMKIIα activity.

LTP is a cellular model of synaptic plasticity. We recorded hippocampal CA3‐CA1 LTP, and found that KN93 reduced HFS‐induced LTP (fEPSP slope) in young mice but had little effect on aged mice (young ctrl: 211.2 ± 15.4% vs. KN93: 148.9 ± 11.7%, ***p = 0.001, Paired *t*‐test, *n* = 8 mice for young group; aged ctrl: 146.5 ± 4.1% vs. KN93: 145.4 ± 9.5%, *p* = 0.93, Paired *t*‐test, *n* = 5 mice for aged group; Figure 2i–j). These results indicate that CaMKIIα is involved in regulation of hippocampal LTP in young but not in aged mice.

### 3.4 Effect of VGCC on the expression of TARPγ8 and p‐CaMKIIα

Nemours studies demonstrated that the increased VGCC activity in aging is a key cause for abnormal increase of [Ca^2+^]_i_ and LTP impairment (Thibault & Landfield, 1996; Thibault et al., 2001; Krueger et al., 2017; Moore et al., 2023) as well as downregulation of NMDAR function and expression (Guidi et al., 2015; Kumar, 2015). Based on these studies, we assume that aging‐related LTP changes may be related to the reduced CaMKIIα activity and TARPγ8 expression. To test this, we first confirmed the expression of L‐type VGCC (Cav1.3) on hippocampal neurons in both young and aged mice (Figure S2). Western Blot analysis showed the expression of Cav1.3 in the hippocampus was higher in the aged mice than that of young mice (young: 0.2 ± 0.019 vs. aged: 0.3 ± 0.077, *t* = −3.909, *p* = 0.03, *n* = 4 for each group; Figure S2a,b). Immunohistochemistry analysis also showed the comparable and enriched hippocampal expression of Cav1.3 in both young and aged mice (Figure S2c). Image J was used to analyze the mean optical density (MOD) of Cav1.3 in hippocampal tissue sections with DAB stains. Our results showed that MOD value in the whole hippocampus of aged mice was larger than that of young mice (young: 2.337 ± 0.086 vs. aged: 2.599 ± 0.012, **p = 0.0065, *t*‐test, *n* = 3; Figure S2d‐i). MOD value of Cav1.3 in CA1 region of aged mice was also larger than that of young mice (young: 2.091 ± 0.070, vs. aged: 2.393 ± 0.079, **p = 0.0077, *t*‐test, *n* = 3; Figure S2d‐ii).

We previously demonstrated that D‐AP5, a NMDAR antagonist, largely reduced LTP in young, but had little effect in aged mice (Guo, Zhao, et al. 2017), which suggest that CaMKIIα activity may have similar aging‐related response to LTP in response to D‐AP5.

Our western blot analysis showed that, similar to previous finding, a significant reduction in hippocampal P‐CaMKIIα expression was found in aged mice, and that D‐AP5 reduced hippocampal P‐CaMKIIα expression in young mice, but had no effect on P‐CaMKIIα expression in aged mice (Figure S3). These results support the notion that NMDAR mediated Ca^2+^ influx is responsible for CaMKIIα activation, a critical process for LTP induction, which is however, downregulated in aging and such a downregulation may contribute to aging‐related decline in LTP and cognitive function.

**FIGURE 3:**
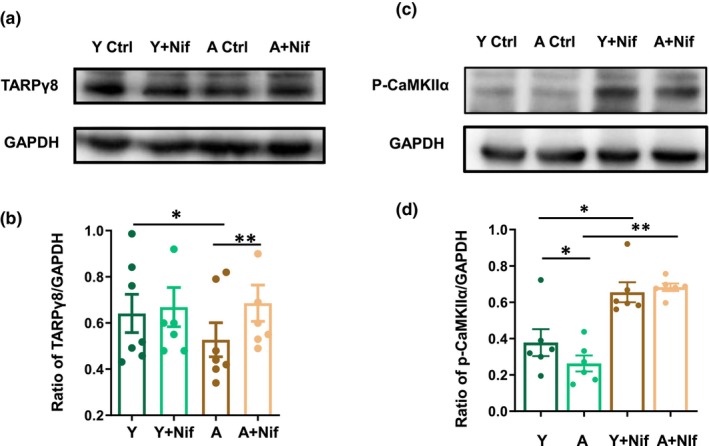
Effect of VGCC blocker on TARPγ8 and p‐CaMKIIα expression. (a) Representative image of TARPγ8 bands (55 kDa) and GAPDH bands (36 kDa) of young and aged mice in the presence or absence of nifedipine (30 μM). (b) The bar graph for the ratio of TARPγ8/GAPDH of young and aged mice (young ctrl vs. young Nif, *p* = 0.57; aged ctrl vs. aged Nif, *p = 0.01, One‐way RM ANOVA followed by post hoc Holm‐Sidak method, *n* = 4 mice for each group). (c) Representative image of p‐CaMKIIα (50 kDa) and GAPDH bands (36 kDa) of young and aging mice in the presence or absence of Nifedipine. (d) The bar graph for the ratio of p‐CaMKIIα/GAPDH of young and aged mice. (young ctrl vs. young Nif, *p = 0.017; aged ctrl vs. aged Nif, **p = 0.009, One‐way RM ANOVA followed by post hoc Holm‐Sidak method, *n* = 4 mice for each group). Y for 3–4 months old mice, A for 22–27 months old mice. Ctrl: Control; Nif: Nifedipine. Data were expressed as mean ± SEM, *p < 0.05; **p < 0.01.

Previous study indicate VGCC blocker was able to restore LTP in aged mice, suggesting that VGCC is a key factor for calcium dyshomeostasis and LTP impairment in aging (Guo, Zhao et al. 2017). Since CaMKIIα‐TARPγ8 signaling was also downregulated in aging, we then explored the relationship between the expression of TARPγ8 and VGCC activity. We found that nifedipine (Nif 30 μM) significantly increased TARPγ8 expression in hippocampal tissues from aged mice but not young mice (aged ctrl: 0.55 ± 0.07 vs. Nif: 0.69 ± 0.08, **p = 0.01; young ctrl: 0.64 ± 0.08 vs. Nif: 0.67 ± 0.09, *p* = 0.57; One‐way RM ANOVA followed by post hoc Holm‐Sidak method, *n* = 7 mice for each group; Figure 3a,b). These results indicate that VGCC negatively modulated TARPγ8 expression in aged mice.

Whether the decreased expression of p‐CaMKIIα in aging is related to VGCC activity has not been reported. We then tested the p‐CaMKIIα expression in the presence of Nif and found that Nif significantly increased the expression of p‐CaMKIIα in hippocampal tissues from both young and aged mice (young ctrl: 0.39 ± 0.12 vs. young Nif: 0.67 ± 0.1, *p = 0.017; aged ctrl: 0.27 ± 0.07 vs. aged Nif: 0.68 ± 0.03, **p = 0.009, One‐way RM ANOVA followed by post hoc Holm‐Sidak method, *n* = 4 mice for each group) (Figure 3c,d). These results suggest that VGCC activity also negatively affected p‐CaMKIIα expression, and inhibition of VGCC activity effectively upregulated CaMKIIα and TARPγ8 expression in aged mice.

### 3.5 Effect of Calpains on the expression level of TARPγ8

**FIGURE 4:**
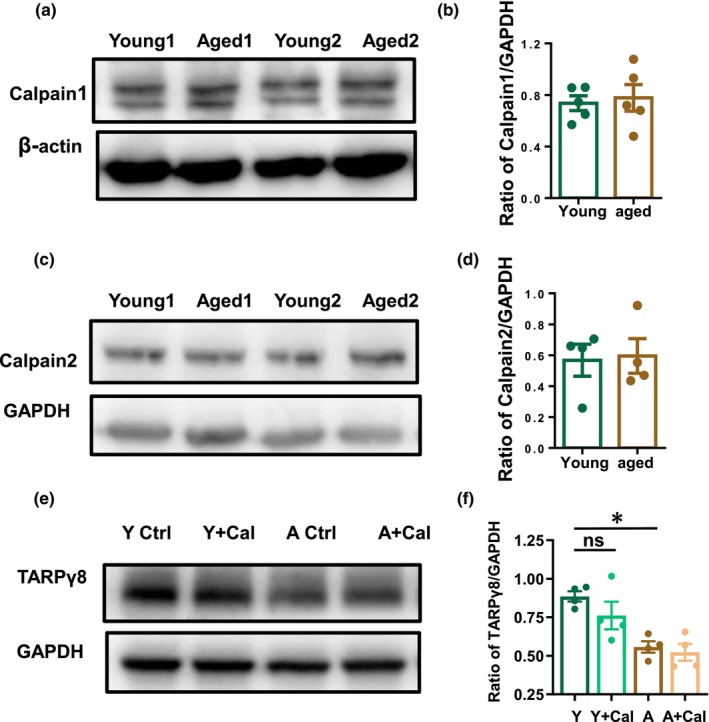
Age‐related changes in Calpain 1 and Calpain 2 expression and the effect of inhibiting Calpain activity on TARPγ8 expression. (a) The representative images of Calpain1 band (82 kDa) and β‐Actin bands (42 kDa) of young and aging mice. (b) The bar graph for ratio of Calpain 1/β‐Actin of young and aged mice (young vs. aged, *p* = 0.535, *t*‐test, *n* = 5 mice for each group). (c) The representative images of Calpain 2 band and GAPDH band of young and aged mice. (d) The bar graph for ratio of Calpain 2/GAPDH of young and aged mice (young vs. aged, *p* = 0.860, *t*‐test, *n* = 4 mice for each group). (e) The representative image of TARPγ8 band and GAPDH band of young and aged mice in the presence or absence of Calpepin. (f) The bar graph for the ratio of TARPγ8/ GAPDH of young and aged mice (young vs. aged, *p = 0.002; young ctrl vs. young Cal, *p* = 0.156, aged ctrl vs. aged Cal, *p* = 0.684, One‐way RM ANOVA followed by post hoc Holm‐Sidak method, *n* = 4 mice for each group). Y for 3–4 months old mice, A for 22–27 months old mice. Ctrl: Control; Cal: Calpepin. Data were expressed as mean ± SEM, *p < 0.05; **p < 0.01; ***p < 0.001.

Aging‐related abnormal increases of intracellular Ca^2+^ may activate nonlysosomal cysteine proteases calpains (Riascos et al., 2014), and consequent degradation of a series of target proteins such as TARPγ8 (Wang, Wang et al. 2016a). To further determine whether calpain is involved in the modulation of TARPγ8 expression in aging, we first examined age‐related changes in hippocampal calpain expression. Our results showed that there was no age‐related change in the expression of hippocampal calpain1 (young: 0.70 ± 0.05 vs. aged: 0.80 ± 0.09, *p* = 0.535, *t*‐test, *n* = 5 mice for each group, Figure 4a,b) or calpain 2 (young: 0.57 ± 0.1 vs. aged: 0.60 ± 0.1, *p* = 0.860, t‐test, *n* = 4 mice for each group, Figure 4c,d). We then investigated the relationship between the expression of TARPγ8 and calpain activity using calpain1 blocker calpeptin (50 μM). Western blot analysis showed that calpeptin had no effect on the expression level of TARPγ8 in young and aged mice (young ctrl: 0.88 ± 0.03 vs. Cal: 0.76 ± 0.09, *p* = 0.156; aged ctrl: 0.56 ± 0.04 vs. Cal: 0.52 ± 0.05, *p* = 0.684, One‐way RM ANOVA followed by post hoc Holm‐Sidak method, *n* = 4 mice for each group, Figure 4e,f).

### 3.6 TARPγ8 knockdown in hippocampi impaired LTP and learning and increased anxiety levels in young mice

**FIGURE 5:**
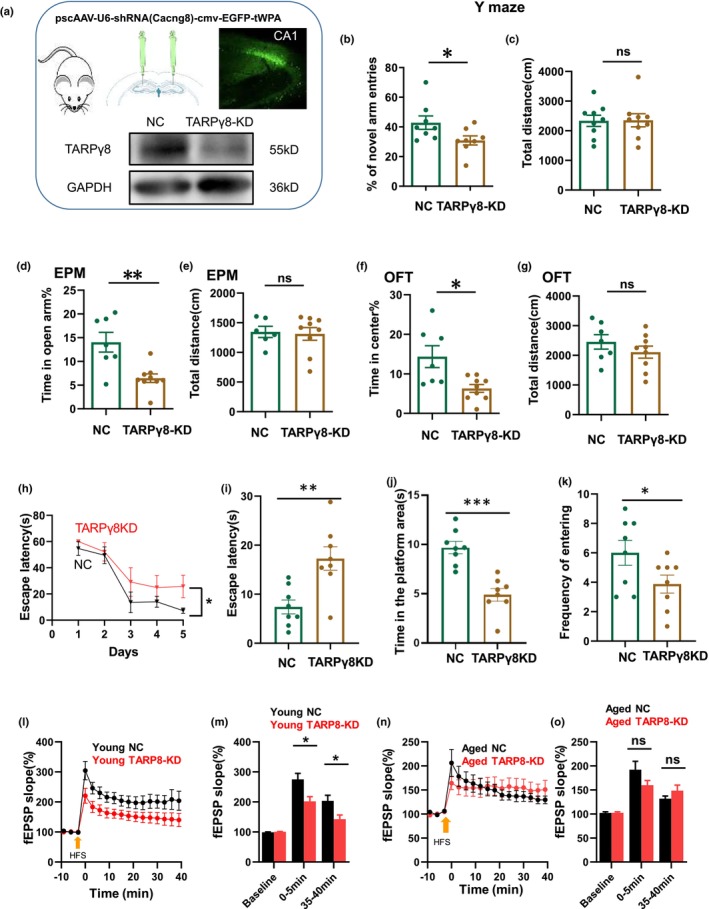
TARPγ8 knockdown affects hippocampal LTP and mouse behavior. (a) A diagram of stereolocation injections in the hippocampal region of a mouse (left top panel); The hippocampal area CA1 in both young and aged mice were injected with a virus pscAAV‐U6‐shRNA(Cacng8)‐cmv‐EGFP‐Twpa (TARPγ8‐KD) or pscAAV‐U6‐shRNA(NC2)‐cmv‐EGFP‐Twpa (Negative control, NC), 300 μm horizontal hippocampal slices were cut and visualized by a two photo microscope (Femtonics) (right top panel). Low panel: Western blot analysis of TARPγ8 expression from mice injected with the NC or TARPγ8‐KD viruses; (b) The percentage of mice (NC and TARPγ8‐KD) entries into the novel arm in Y maze. (c) Total distance of mice traveled in Y maze. (d) The percentage of time that mice spent in open arm in EPM. (e) Total distance of mice traveled in EPM. (f) The percentage of time that mice spent in the center in OFT. (g) Total distance of mice traveled in OFT. (h) Training consisted of 5 days (D1––D5) in which mice (NC, *n* = 8; TARPγ8‐KD, *n* = 8) were placed at random locations around the edge of the pool and allowed to swim for 60 s or until they found the platform. (i) Escape latency measured in probe test for spatial memory. (j) Time spent in the platform area. (k) Frequency of mice entering the platform area. (l) The time‐effect curves of the field EPSP (fEPSP) slope before and after HFS in young mice. (m) Histogram gives the HFS‐induced LTP magnitude for early LTP peak state (average over 0–5 min post HFS) and steady state (average over 35–40 min post HFS), for slices from NC group (*n* = 7 slices from 4 mice) or TARPγ8‐KD in young mice (*n* = 6 slices from 4 mice). (n) The time‐effect curves of fEPSP slope before and after HFS in aged mice. (o) Histogram gives the HFS‐induced LTP slope for early LTP peak state (average over 0–5 min post HFS) and steady state (average over 35–40 min post HFS), for slices from NC group (*n* = 6 slices from 4 mice) or TARPγ8‐KD in aged mice (*n* = 7 slices from 7 mice). Student's *t*‐test was used for comparisons between slices from NC or TARPγ8‐KD mice. Data were given as mean ± SEM. *, *p* < 0.05; **, *p* < 0.01.

Previous studies identified the hippocampus‐enriched TARPγ8, but not TARPγ2/3/4, as a critical CaMKII substrate for LTP (Park, Chavez, et al., 2016). To verify the role of TARPγ8 on cognitive function, PscAAV‐U6‐shRNA (Cacng8)‐cmv‐EGFP‐Twpa or pscAAV‐U6‐shRNA (NC2)‐cmv‐EGFP‐Twpa viruses were bilaterally injected into the hippocampal CA1 of mice to knockdown TARPγ8 (TARPγ8‐KD mice), the expression of TARPγ8 was confirmed by western blotting 3 weeks later (Figure 5a). Mice were then subjected to behavioral experiments. Compared with mice injected with negative shRNA control (NC), Y‐maze study showed the reduced percentage of novel arm entries (NC: 42 ± 4% vs. TARPγ8‐KD: 31 ± 3%, *p = 0.046, *t*‐test, *n* = 8 mice for each group) without affecting the total distance that mice traveled in maze (NC: 4235 ± 187 vs. TARPγ8‐KD: 2355 ± 220, *p* = 0.947, *t*‐test, *n* = 9 mice for each group, Figure 5b,c).

EPM showed TARPγ8‐KD mice had the reduced percentage of the time spent in open arm (NC: 14.0 ± 2.1% vs. TARPγ8‐KD: 6.4 ± 0.9%, *p = 0.01, *t*‐test, *n* = 7–9 mice for each group) without changing the total distance that these mice traveled (NC: 1345 ± 93 vs. TARPγ8‐KD: 1311 ± 107, *p* = 0.828, *t*‐test, *n* = 7–9 mice for each group, Figure 5d,e).

OFT showed the reduced percentage of the time in center (NC: 14.3 ± 2.7% vs. TARPγ8‐KD: 6.3 ± 0.9, *p = 0.027, *t*‐test, *n* = 7–9 mice for each group) without changing the total distance in TARPγ8‐KD mice (NC: 2453 ± 245 vs. TARPγ8‐KD: 2106 ± 202, *p* = 0.289, *t*‐test, *n* = 7–9 mice for each group, Figure 5f,g). These results indicated the TARPγ‐8 KD mice had decreased capability in learning and increased anxiety.

Mice were trained to find a hidden escape platform over the course of 5 days and the time required to reach the platform (latency) was used as a measure of spatial learning. NC mice exhibited a significant reduction in latency across days of training (NC: Day1 vs. Day 5, *p* = 0.005, t = 6.067, *n* = 8). TARPγ8‐KD mice showed a tendency of reduction in latency across days of training without statistical significance. There was a significant difference in latency between the two groups (NC‐Day5: 7.3 ± 2.1 s vs. TARPγ8‐KD‐Day 5: 25.7 ± 8.5 s, *p* = 0.03, t = 2.418, Figure 5h). To assess spatial reference memory, probe trials were conducted on day 7. The results suggest the NC group had already formed a memory for the platform location. Conversely, the TARPγ8‐KD mice spend more time to search for the platform (Figure 5i–k).

The LTP in the hippocampal CA1 was decreased in TARPγ8‐KD mice compared with age‐matched NC mice, but was no different from aged mice (young NC: 203.5 ± 18.5 vs. TARPγ8‐KD: 142.6 ± 14.1, *p = 0.016, Paired *t*‐test, *n* = 8–12 mice for young group; aged WT: 131.6 ± 5.6 vs. TARPγ8‐KD: 148.4 ± 11.6, *p* = 0.218, *t*‐test, *n* = 10–12 mice for aged group, Figure 5l–o). These results demonstrated that TARPγ‐8 deficiency impaired synaptic plasticity.

### 3.7 Effect of exogenous expression of human TARPγ8 on synaptic plasticity in aged mice

**FIGURE 6:**
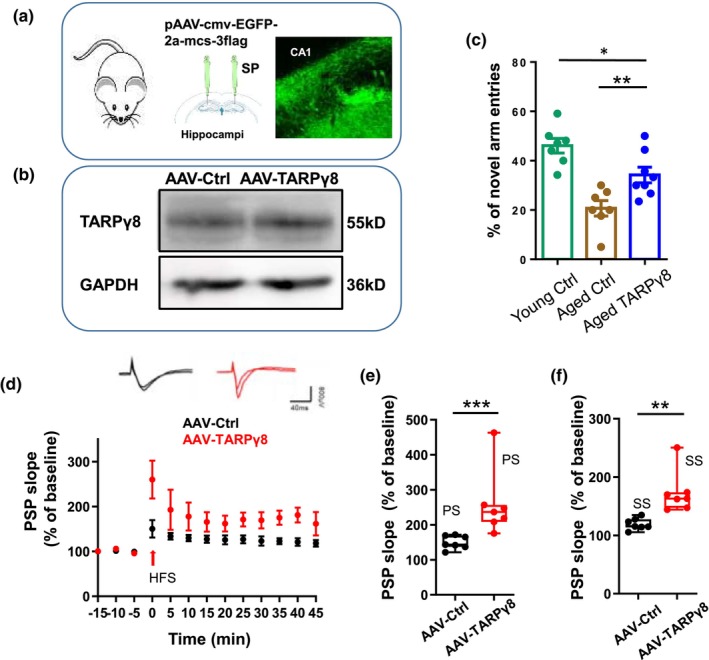
Effects of exogenous expression of human TARPγ8 on cognitive function and hippocampal LTP in aged mice. (a) A diagram of stereolocation injections in the hippocampal region of a mouse; The hippocampal area CA1 in an aged mouse was injected with a control plasmid (pAAV‐cmv‐egfp‐2a‐mcs‐3flag), three weeks later, 300 μm horizontal hippocampal slices were cut and visualized by a two photo microscope (Femtonics) (right top panel). (b) Western blot analysis on the TARPγ8 expression from mice injected with the control or TARPγ8 plasmid; (c) the behavioral experiments of Y‐maze showed that the percentage of entering novel arm for aged mice injected with TARPγ8 and control plasmid (TARPγ8: 0.34 ± 0.03 vs. ctrl: 0.19 ± 0.15, *p = 0.022, *t*‐test, *n* = 8 mice per group); (d) The time‐effect curves of the field EPSP (fEPSP) slope of before and after HFS. The stimulus electrode was located stratum radiatum and the recording electrode was in stratum pyramidale of CA1 in mouse ventral hippocampal slices. CA1 population synaptic potential (PSP) measurements: The PSP amplitude was taken as difference between the baseline and the PSP. The slope was taken from the start of the PSP and to half‐way the downward phase. The PSP slope normalized to average of the PSP slope over 10 min pre‐HFS baseline from aged mice injected with AAV‐ctrl plasmid (*n* = 6 slices from 4 mice) and aged mice injected with AAV‐TARPγ8 plasmid (*n* = 7 slices from 4 mice). The insets gave 40 ms traces taken from representative experiments illustrating the average PSP 5 min prior to HFS and 35–40 min post‐HFS. The left pair of traces is from an aged mouse injected with AAV‐ctrl plasmid; the right pair of traces is from an aged mouse injected with AAV‐TARPγ8 plasmid. (e, f) Histogram gives the HFS‐induced LTP magnitude (percentage increase of PSP slope relative to baseline) for early LTP peak state (average over 0–5 min post HFS) and steady state (average over 35–40 min post HFS), for slices from aged mice injected with AAV‐Ctrl plasmid or AAV‐TARPγ8 plasmid. Student's *t*‐test was used for comparisons between slices from AAV‐Ctrl and AAV‐TARPγ8 injected aged mice. Data were given as mean ± SEM. *p < 0.05; **p < 0.01; ***p < 0.001.

Previous studies have shown impaired hippocampal CA1 LTP in aging (Guo, Zhao et al. 2017). To test whether TARPγ8 can improve the impaired LTP and learning at the whole animal level in aged mice, we exogenously expressed human TARPγ8 in the hippocampi of aged mice to determine the effect of the TARPγ8 in these mice. The pAAV‐cmv‐egfp‐2a‐Cacng8‐3flag (AAV‐TARPγ8) and the control plasmid pAAV‐cmv‐egfp‐2a‐mcs‐3flag (AAV‐Ctrl) were injected into the hippocampal CA1 region (Figure 6a). Western blot data showed that we have successfully overexpressed the TARPγ8 protein (Figure 6b). The behavioral experiments (Y maze) showed that the proportion of entering the novel arm in the aged mice injected with AAV‐TARPγ8 was significantly higher than that of the aged control mice (TARPγ8: 0.34 ± 0.03 vs. ctrl: 0.21 ± 0.02, *p = 0.012, *t*‐test, *n* = 7 and 8 mice for the aged mice injected with AAV‐ctrl and AAV‐TARPγ8 plasmid, respectively, Figure 6c). The normalized slope of LTP from mice injected with AAV‐TARPγ8 plasmid was significantly higher than that of mice injected with control plasmid (Figure 6d). The normalized peak slope of fEPSP at 0–3 min after HFS from mice injected with AAV‐TARPγ8 was significantly higher than that of mice injected with AAV‐control plasmid (AAV‐TARPγ8: 236.8(211, 252.6) %, *n* = 7 slices from 5 mice vs. AAV‐control: 143.5(138.3168.3) %, *n* = 7 slices from 4 mice, ***p = 0.001, Mann–Whitney Rank Sum Test). Similarly, the normalized steady‐state slope at 35–40 min after HFS from mice injected with AAV‐TARPγ8 was higher than that of mice injected with AAV‐control plasmid (AAV‐TARPγ8: 163(151.1172.7) %, *n* = 7 slices from 5 mice vs. AAV‐ctrl 115.6 (114.5, 126.4) %, *n* = 7 slices from 4 mice, **p = 0.002, Mann–Whitney Rank Sum Test) (Figure 6e,f). These results indicated that exogenous expression of human TARPγ8 could reverse the impaired synaptic plasticity and memory function in aged mice.

## 4 DISCUSSION

In this study, we found that (1) age‐dependent decrease in TARPγ8 expression was correlated with the decline in p‐CaMKIIα and dependent on CaMKIIα activity; (2) age‐related decline in both TARPγ8 and p‐CaMKIIα levels was regulated by VGCC activity; (3) knockdown of TARPγ8 in hippocampi impaired LTP and cognitive function in young mice; (4) exogenous expression of TARPγ8 in aged mice restored the aging‐related impairment of LTP and cognitive function. Our results indicate that TARPγ8 expression is mediated by p‐CaMKIIα level and governed by VGCC activity. Aging‐related VGCC activation negatively mediated CaMKIIα‐ ARPγ8 signaling and contributed to cognitive impairment in aging.

### 4.1 Aging‐related decrease in TARPγ8 expression

TARPγ8 is enriched in the hippocampus (Dohrke et al., 2020; Straub & Tomita, 2012), where it controls AMPAR expression at the synapses dominated by the Schaffer lateral (Yamasaki et al., 2016). TARPγ8 mediated not only AMPAR membrane insertion but also the opening time of the AMPAR channel (Carrillo et al., 2020). The mechanism by which TARPγ8 regulates AMPAR membrane expression may be related to the glycosylation of TARPγ8 (Zheng, Chang et al., 2015). Recent studies using a cry‐electron microscopy approach have shown structural evidence for the binding of TARPγ8 to heterozygous GluA 1/2 AMPAR (Herguedas et al., 2019). Tetramer GluA and TARPs are assembled in the endoplasmic reticulum (Schwenk, Boudkkazi, et al., 2019). Aging‐related decrease in TARPγ8 expression likely involved the disturbance of membrane expression of GluA, synaptic transmission and plasticity and, consequently, the reduced cognitive function in aging.

### 4.2 The TARPγ8 expression was dependent on CaMKIIα activity

CaMKIIα is essential for synaptic plasticity and learning (Cai et al., 2021). Studies have shown that CaMKIIα mediated TARPγ8 phosphorylation, is necessary for the induction of LTP (Lisman et al., 2012; Park, Chávez, et al., 2016). CaMKIIα also mediated the phosphorylation of other members of the family of the TARP (TARPγ2) (Rouach et al., 2005) and AMPAR (Lee et al., 2010), but the phosphorylations of these two receptors were not essential for LTP (Granger et al., 2013; Park, Chavez, et al., 2016). In addition, CaMKIIα has been reported to involve LTP mediated by dopamine D1/D5 receptors in hippocampal CA1 pyramidal neurons and plays a key role in long‐term memory performance (Shetty, Sajikumar, et al., 2017; Śliwińska et al., 2020).

Theoretically, VGCC‐dependent [Ca^2+^]_i_ increase should lead to CaMKIIα activation, the present study showed the notably decreased CaMKIIα phosphorylation in aging, perhaps related to increased VGCC activity and associated dysregulation of calcium in aging (Palomer et al., 2016; Zhao et al., 2010).

Inhibition of CaMKIIα activity significantly reduced the expression of TARPγ8 in hippocampi from young and aged mice, suggesting that the TARPγ8 expression is dependent on the activity of CaMKIIα and the dramatic decline in TARPγ8 expression in young mice mimics an age‐related change in TARPγ8. Inhibition of CaMKIIα activity also reduced TARPγ8 expression level of aged mice, but to a lesser extent. Thus, TARPγ8 expression level appears to be a useful index for brain function in aging.

Whereas our data indicate the regulation of CaMKIIα on TARPγ8 expression, other research suggests that the CaMKII/TARPγ‐8 pathway was involved in depression (Sakai et al., 2021). CaMKIIα‐TARPγ8 signaling was involved in impaired cognitive function in depression, over‐expression of TARP‐γ8 reverses chronic stress‐induced depressive‐like behaviors and attenuation of AMPARs‐mediated neurotransmission. Conversely, the knockdown of TARP‐γ8 in excitatory neurons prevents the rapid antidepressant effects of ketamine. (Xue et al., 2023).

Aging‐related change in TARPγ8 expression appeared not to be associated with calpain activity, despite calpain being known to degrade AMPAR (Yuen, Gu, et al., 2007), modify TARPγ8 expression (Wang et al., 2016a) and increase in aging (Riascos et al., 2014). Previous studies also showed that calpain1 is required for induction of LTP induced by theta‐burst stimulation in hippocampal CA1 field in young rodents (Briz, Baudry, et al., 2017; Zhu, Liu et al., 2015) and a calpain inhibitor restored the synaptic function in animal models of AD (Trinchese et al., 2008). In this study, inhibition of calpain1 activity had no effect on TARPγ8 level in either young or aged mice, suggesting that calpain1 activity is not involved in TARPγ8 expression. It was reported that CaM‐binding proteins, CaMKII may be the substrates of calpain 2 (Chang et al., 2015; Tremper‐Wells & Vallano, 2005a). Given that TARPγ8 expression is dependent on p‐CaMKIIα level, aging‐related changes in calpain 2 expression or activity may cause the decline of p‐CaMKIIα level and consequently, the downregulation of TARPγ8 expression. However, we did not observe altered expression level in either Calpain 1 or Calpain 2 in aging which make this hypothesis unlikely.

### 4.3 Aging‐related changes in TARPγ8 expression are mediated by VGCC

Aging is associated with increased VGCC expression and [Ca^2+^]_i_ (Thibault et al., 2007) (Thibault & Landfield, 1996). The increased VGCC density is a main driver for calcium dysregulation and impaired learning related to aging and underlies the vulnerability of neurons to age‐associated neurodegenerative conditions. Interestingly, overexpression of L‐type VGCC, CaV1.3 in young mice is sufficient to drive changes in neuronal physiology such as an increase in the magnitude of the post burst afterhyperpolarization and a deficit in spatial learning and memory, similar to those observed in aged animals (Krueger et al., 2017; Moore et al., 2023).

Blocking VGCC completely prevents age‐related decline in TARPγ8, verifying the VGCC‐mediated downregulation of TARPγ8. Although the mechanism related to VGCC‐mediated decline of TARPγ8 remains to be further determined, the evidence of VGCC blocker increasing p‐CaMKIIα levels in both young and aged mice provides a clue that VGCC activation caused downregulation of p‐CaMKIIα levels, which contribute to the restoration of the TARPγ8 expression in aged mice. Interestingly, VGCC blockade enhanced LTP in aged mice (Guo et al., 2017) and diminish cognitive loss and preserve endothelial function during diabetes mellitus (Jain et al., 2016). Furthermore, a recent study also demonstrated that VGCC blocker prevents cognitive impairment in an aged mouse model of sporadic Alzheimer's Disease (Ahmed et al., 2021). It would be interesting to determine whether the beneficial effect of VGCC blockers on cognitive function in these animal models is related to enhanced expression of p‐CaMKIIα and TARPγ8.

### 4.4 TARPγ8 knockdown impaired LTP, and memory performance, and increased anxiety‐like behavior

TARPγ8 plays a crucial role in regulating the function and trafficking of AMPA receptors in the brain, and AMPA receptors are important for synaptic transmission and plasticity, including LTP and learning processes. TARPγ8 knockdown disrupted the proper functioning of AMPA receptors, leading to impairments in LTP and subsequently affecting learning and memory processes. Thus, aging‐related reduction in TARPγ8 may be also a key factor for aging‐related impairment of LTP and cognitive function.

In addition to cognitive function impairment, our data indicate that the increased anxiety in TARPγ8 knockdown mice was in agreement with the previous study reported by Wan‐Jun Bai et al., who reported that adolescent TARPγ8 knockout mice exhibited ADHD‐like behaviors, including hyperactivity, impulsivity, anxiety, impaired cognition, and memory deficits (Bai et al., 2022).

The increase in anxiety‐like behaviors suggests an involvement of TARPγ8 in regulating emotional states. The exact mechanisms underlying this increase in anxiety are not fully understood, it is likely that the disruption of AMPA receptor function resulting from TARPγ8 knockdown may alter synaptic transmission in brain regions associated with anxiety regulation. This may also explain the consequent poor memory performance. A validated all‐in‐one test is required to distinguish the role of TARPγ8 in these behavioral effects (Ennaceur et al., 2011).

### 4.5 Exogenous overexpression of TARPγ8 restored aging‐related reduction of LTP and learning

The activation of CaMKIIα‐mediated TARPγ8 phosphorylation is required for induction of LTP and the mutation at the phosphorylation site of TARPγ8 shows significant LTP and learning and memory impairment (Park, Chávez, et al., 2016). Aging is associated with impaired LTP (Guo et al., 2017), reduced CaMKIIα activity, and TARPγ8 expression (Kelly et al., 2014). Exogenous expression of TARPγ8 in the hippocampus increased LTP in aged mice, confirming the importance of TARPγ8 for LTP induction. Y maze effectively reflects the environment recognition memory and is commonly used to test the spatial working memory of rodents in the study (Kraeuter, Guest, et al., 2019). The aged mice injected with the TARPγ8 plasmid also show improved spatial working memory compared to those mice injected with the control plasmid, which further underlines the essential role of TARPγ8 on LTP and learning. Interestingly, a recent study showed that overexpression of TARPγ8 reverses chronic stress‐induced depressive‐like behaviors and attenuation of AMPARs‐mediated neurotransmission (Xue et al., 2023). The levels of overexpression and the influence of stress may be crucial to explain these differences.

In conclusion, our results show that the aging‐related decrease of TARPγ8 expression mediated by reduced p‐CaMKIIα and increased VGCC activity leads to aging‐related impairment of synaptic plasticity, cognitive function, and emotional stability.

## AUTHOR CONTRIBUTIONS

J.Z., M.L., Q.H. and P.D. performed research, analyzed data; S.J., J.L., S.L., X.R., Z.Z., J.D. and Y.Y. analyzed data; P.C. initiated project, designed and supervised research; C.L. designed research, analyzed data, wrote and revised paper.

## CONFLICT OF INTEREST STATEMENT

The authors declare no completing interests.

### OPEN RESEARCH BADGES

This article has earned an Open Data badge for making publicly available the digitally‐shareable data necessary to reproduce the reported results. The data is available at [[insert provided URL from Open Research Disclosure Form]].

## Supporting information


Figure S1.

## Data Availability

The data that support the findings of this study are available on request from the corresponding author.
