# Supplementary figures and images for: CaMKIIα‐TARPγ8 signaling mediates hippocampal synaptic impairment in aging
Source: Aging Cell. 2024 Oct 8;24(1):e14349. doi: 10.1111/acel.14349 (PMC11709088; doi:10.1111/acel.14349)

Supplement Fig 1

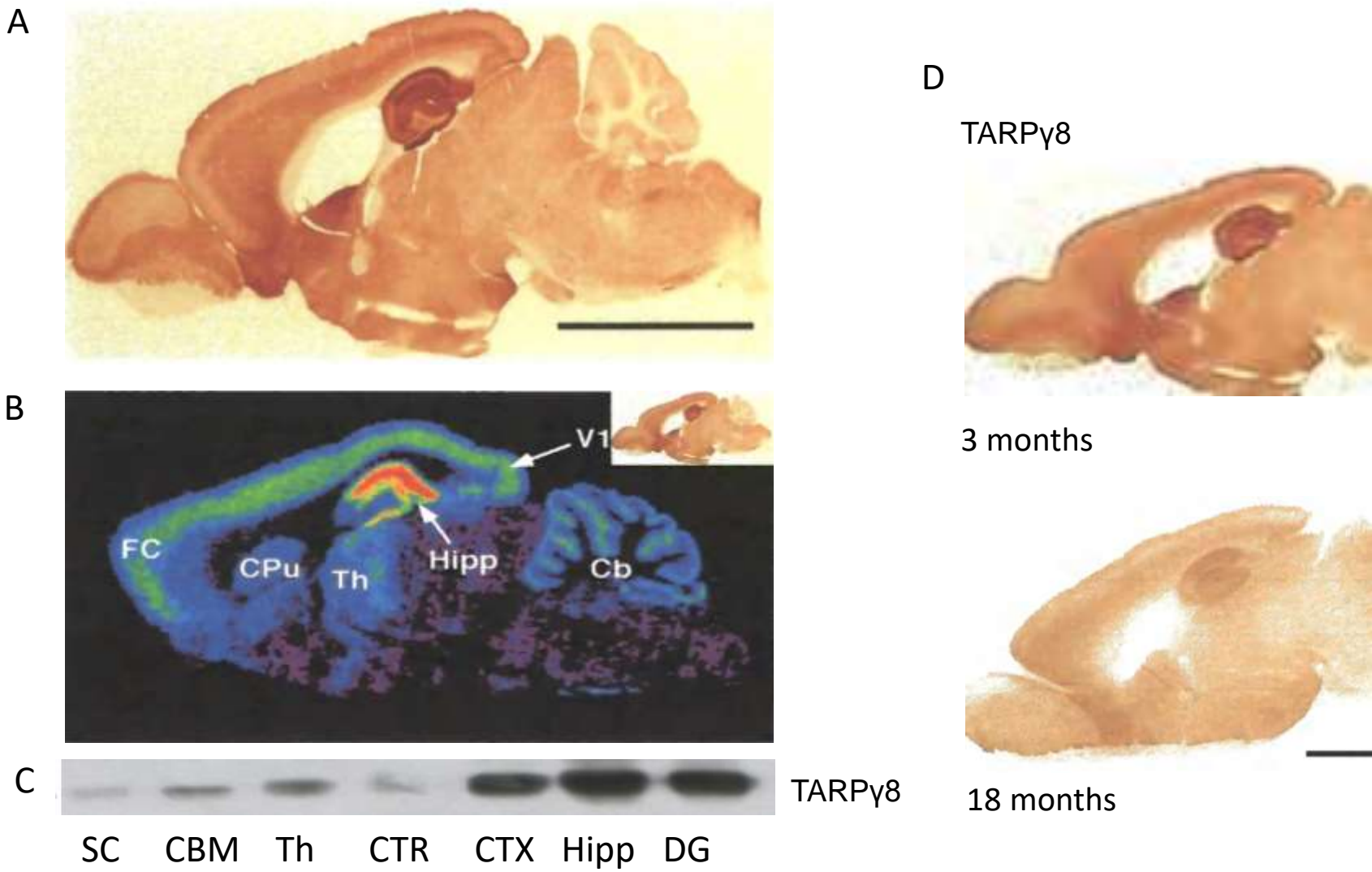

Supplement Fig2

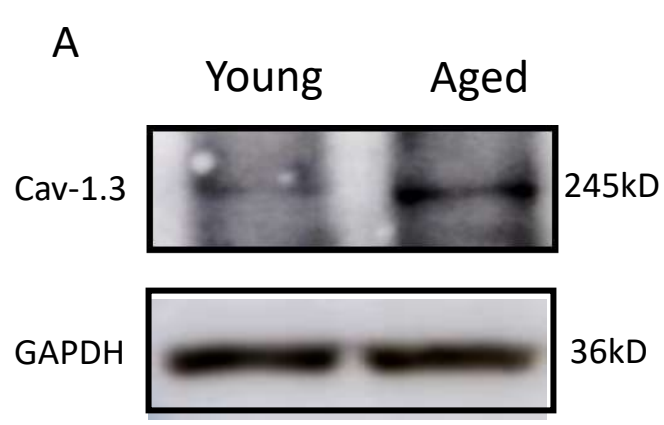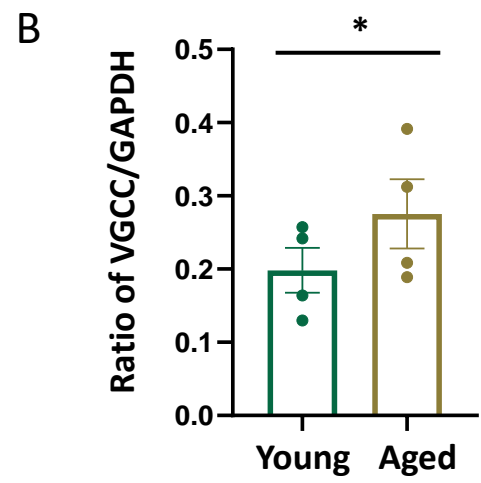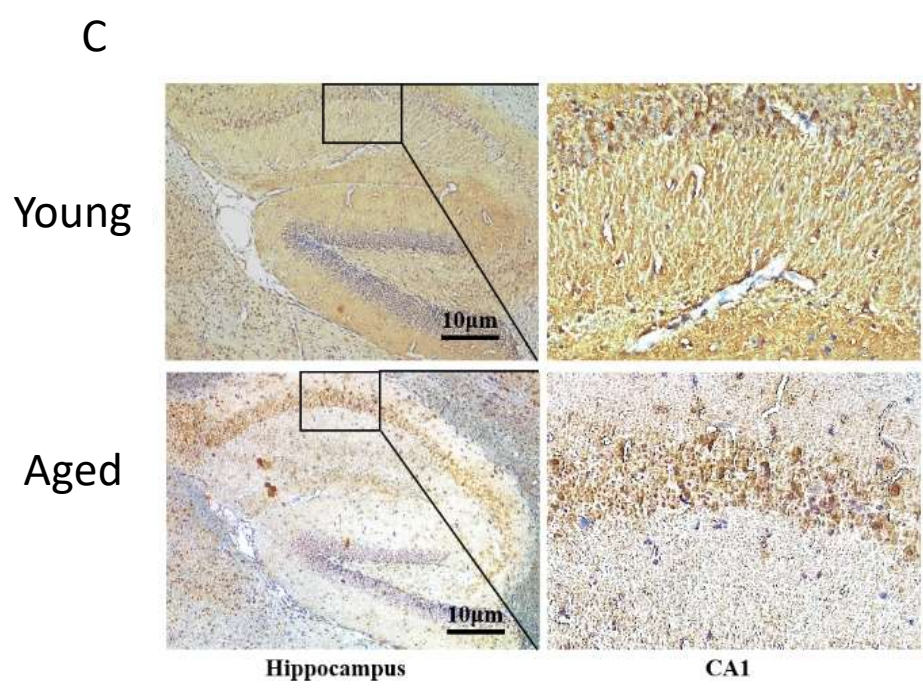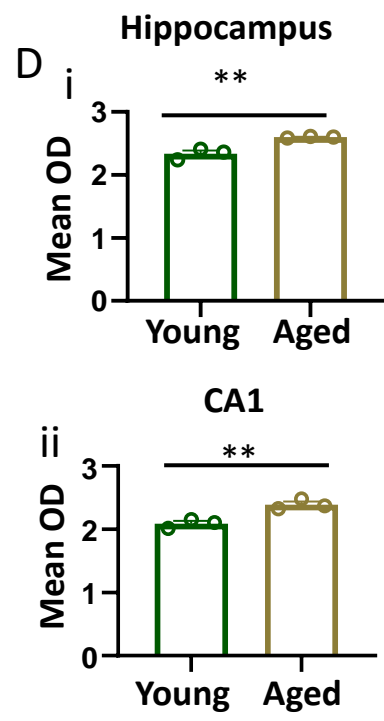

Supplement Fig3

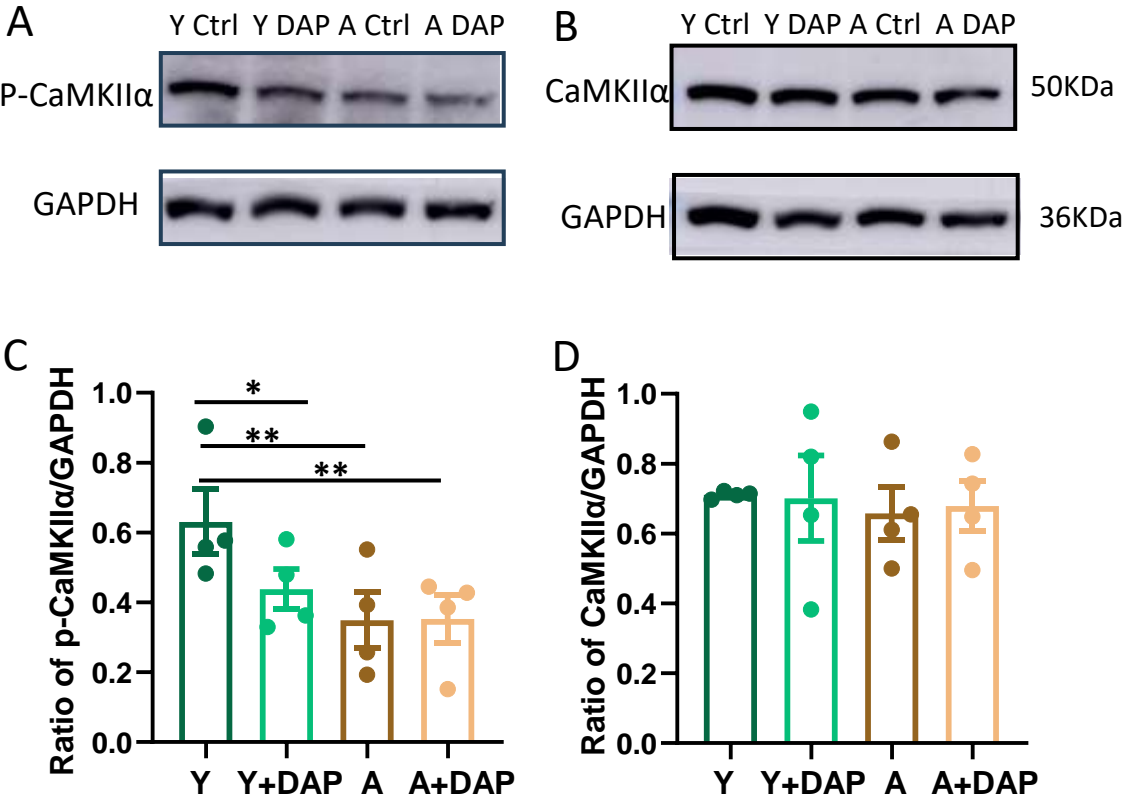

Supplement: Supplementary file 1 — Figure S1. [file ACEL-24-e14349-s001.pdf]
